# Self‐Healing and Shape Memory Effects in Gold Microparticles through the Defects‐Mediated Diffusion

**DOI:** 10.1002/advs.201700159

**Published:** 2017-07-07

**Authors:** Oleg Kovalenko, Christian Brandl, Leonid Klinger, Eugen Rabkin

**Affiliations:** ^1^ Department of Materials Science and Engineering Technion – Israel Institute of Technology 32000 Haifa Israel; ^2^ Institute for Applied Materials IAM‐WBM Karlsruhe Institute of Technology D‐76344 Eggenstein‐Leopoldshafen Germany

**Keywords:** atomic force microscopy, metal nanoparticles, nanoindentation, shape memory effect, surface diffusion

## Abstract

Some metal alloys subjected to irreversible plastic deformation can repair the inflicted damage and/or recover their original shape upon heating. The conventional shape memory effect in metallic alloys relies on collective, or “military” phase transformations. This work demonstrates a new and fundamentally different type of self‐healing and shape memory in single crystalline faceted nano and microparticles of pure gold, which are plastically deformed with an atomic force microscope tip. It is shown that annealing of the deformed particles at elevated temperatures leads to nearly full recovery of their initial asymmetric polyhedral shape, which does not correspond to global energy minimum shape. The atomistic molecular dynamic simulations demonstrate that the shape recovery of the particles is controlled by the self‐diffusion of gold atoms along the terrace ledges formed during the particles indentation. This ledge‐guided diffusion leads to shape recovery by the irreversible diffusion process. A semiquantitative model of healing developed in this work demonstrates a good agreement with the experimental data.

## 1 Introduction

Self‐healing materials are able to recover their structural integrity after inflicted mechanical damage. The design of artificial smart self‐healing materials is inspired by the numerous examples of biological systems exhibiting the ability of self‐healing after being wounded. While most of the research on biomimetic self‐healing materials is focused on polymers and polymer‐based nanocomposites,^1^ several concepts of self‐healing in metallic materials have recently emerged.^2^ These concepts include the stress‐ and capillary‐driven segregation and precipitation of the second phase in the creep induced cavities or at the crack tips,^3^, ^4^ incorporation of the shape memory alloy (SMA) wires in the metal matrix composite,^5^ and the use of encapsulated solders.^6^ All these concepts rely on some kind of phase transformation (i.e., melting, precipitation, diffusionless martensitic transformation) in the multicomponent, multiphase composite materials. Yet no comparable experimental observations of self‐healing in pure metals are available in the literature; although the theoretical possibility of crack self‐healing in pure metal through diffusionless disclination–crack interaction is predicted by molecular dynamics (MD) simulation.^7^

Contrary to self‐healing, the shape memory effect describes the recovery of the original shape of the plastically deformed alloys upon heating, without reference to their internal microstructure. In SMAs, the shape memory is achieved through the twins‐mediated plasticity and reversible martensite–austenite phase transformation.^8^ Fundamentally different atomistic mechanisms are responsible for shape recovery in polymer materials.^9^ The capillary‐driven self‐diffusion in solids can produce a number of effects exhibiting some features of self‐healing and shape memory effects, or of their combination. This process can heal the artificially introduced perturbations of surface topography, recovering the original shape minimizing the total surface energy of the system. For example, a sinusoidal perturbation of initially planar surface decays exponentially with time during annealing at elevated temperatures, and the appropriate diffusion coefficient can be extracted from the kinetics of this decay.^10^ At the final stage of the healing process the original planar surface morphology corresponding to the minimum of surface energy is restored. Also, partial healing of the cracks stimulated by the curvature‐driven diffusion was observed in metals,^11^ ceramics,^12^ and even ice.^13^ This kind of healing, however, is never complete because of the fast decrease of the driving force for diffusion with decreasing curvature of the crack tip.

In the present work, we observed the combined self‐healing and shape memory effects in the faceted asymmetric single crystalline Au nanoparticles attached to a sapphire substrate. The particles were plastically deformed by a hard diamond atomic force microscope (AFM) tip, and restored their original asymmetric shape upon annealing. The most intriguing fact in the observed phenomenon was that the restored particle shapes did not correspond to any (even metastable) energy minimum.

## 2 Results and Discussion

### 2.1 Experimental Observations

The Au particles on sapphire substrate were obtained by the solid state dewetting of lithographically prepatterned 30 nm thick film,^14^ as described previously.^15^ The obtained particles were single crystalline and exhibited irregular truncated polyhedral shapes with only {100} and {111} facets present, and [111] axis being normal to the substrate. The in‐plane orientation of the particles was quasi‐random, with some preference for the alignment of the densely packed rows of the {111} plane of Au and of the basal plane of sapphire: Au[110]||Al_2_O_3_ $\left[10\bar{1}0\right]$ and, to a lesser extent, Au$\left[\bar{2}11\right]$||Al_2_O_3_ $\left[10\bar{1}0\right]$.^16^ The lateral size of the particles varied from several hundred nanometers to one micrometer, while their height varied from 100 up to 350 nm. Though the {100} and {111} facets correspond to the surfaces of the lowest energy in gold,^15^, ^17^ the shapes of the particles were far from the equilibrium Wulff–Winterbottom shape corresponding to the minimum of surface energy for the particle of constant volume.^18^, ^19^ This is because the energy barrier associated with the normal movement of the facets in the single crystalline, defect‐free particles is prohibitively high for the particles larger than few nanometers in size,^20^ and most particles inherit the low aspect ratio of the thin film they were formed from. Recently, it was demonstrated that the equilibration of Au and Fe particles can be accelerated by plastic deformation with the AFM tip.^15^, ^21^

**Figure 1:**
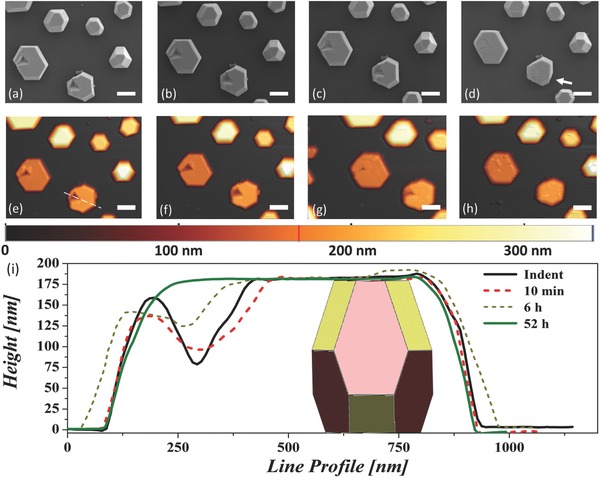
Self‐healing and shape memory in the indented gold particles: a–d) the HR‐SEM and e–h) AFM images of the typical particles indented with the maximum load of 32 µN: a,e) after indentation, b,f) after annealing for 10 min, c,g) after annealing for 6 h, and d,h) after annealing for 52 h (cumulative annealing times are given). The scale bars are 500 nm. i) The AFM topography profiles taken along the line marked in panel (e) are given for all annealing times. The equilibrium shapes of gold crystals of the same height on sapphire are given in panel (i) for comparison.

**Figure 2:**
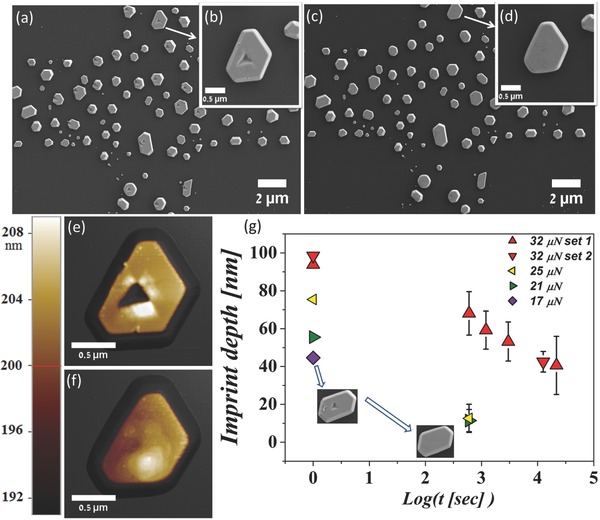
The HR‐SEM and AFM images of the particles a,b,e) indented with the maximum load of 25 µN and c,d,f) after annealing for 10 min. The dependence of the indent depth on annealing time averaged for all studied particles is shown in panel (g). The particles indented with the lowest load of 17 µN have fully recovered after the shortest annealing time of 10 min at 873 K.

We employed the AFM‐based nanoindentation to produce controlled plastic deformation of the particles. Though the AFM‐based indentation is inferior to the depth‐sensing indentation in terms of the quantitative characterization of the indentation modulus and hardness, it provides higher load accuracy in the region of low loads (below 50 µN), and allows precise positioning of the indents. The Au particles were deformed with the maximum loads of 17, 21, 25, and 32 µN. The depths of the obtained indents varied significantly for the same load, exhibiting direct correlation with the aspect ratio of the particles (see Figure S1 of the Supporting Information).^22^ This is related to the efficiency of the side surfaces of the particle to absorb dislocations generated during indentation.^22^, ^23^ The dislocations glide geometry in the particles with low aspect ratio does not allow a direct egress of the dislocations to the side surfaces, thus increasing the probability of formation of sessile dislocation structures inside the particle exerting the back‐stress on the indenter and increasing the particle hardness. The patterning of the thin film prior to dewetting allowed an easy identification of the individual indented particles after consecutive heat treatments at 873 K. The high‐resolution scanning electron microscopy (HR‐SEM) and AFM images illustrating indents healing upon consecutive heat treatments in several typical indented particles are presented in **Figures** **1** and **2**. The Figure 1 demonstrates that full recovery of the indents produced with the highest load of 32 µN was observed after the longest annealing time of 52 h. The annealing restored the heights and the lateral sizes of the two particles presented in Figure 1 with the accuracies of ±1 nm and ±10 nm, respectively (see Figure 1i), which correspond to less than 1% of their respective dimensions. A minor variation of the shape of the side facets was observed for the particle 2 (marked by an arrow in Figure 1d). It is remarkable that both particles returned to their initial, nonequilibrium shapes. This is illustrated on the linear AFM topography profiles in Figure 1i, in which the equilibrium shape of the Au particle of the same height is shown for comparison.^15^, ^17^

The Figure 2 demonstrates that decreasing the maximum indentation load leads to a significant shortening of the healing time: the particle indented with the maximum load of 25 µN (see Figure 2a,b,e) has nearly recovered its original shape after annealing for just 10 min (Figure 2c,d,f). The dependencies of the imprints depths on annealing time for all studied particles are shown in Figure 2g. It should be noted that the healing rate of the indents is highly nonlinear in time. Indeed, the average ratio of the volumes of the indents produced with the highest (32 µN) and the lowest (17 µN) loads is about five (see Figure S1 of the Supporting Information), whereas the corresponding healing times differ by more than two orders of magnitude.

The healing of the indents and the recovery of the original nonequilibrium shape of the particles observed in this work raises two important questions: (i) why the diffusion processes in the particles, which are fast enough for a full healing of the indents, cannot cause the overall shape of the particles to evolve closer to the thermodynamic equilibrium? It should be noted here that higher plastic strains and higher annealing temperature did result in equilibration of the Au particles of similar shape and dimensions.^15^ Apparently, not only the self‐diffusion coefficient on the surface of the particle but also the temperature‐dependent internal processes in the deformed particle and the relative importance of different diffusion paths play a role in shape evolution of the particle. (ii) How the Au atoms diffusing in‐ or on the particles know that they have to arrive at the indent and fill it? Indeed, the conservative dislocations motion during particles indentation leads to the sideward material flow and increase of the lateral dimensions of the particles, compensating for the material deficit created in the indented zone.^22^ This material accumulated at the side facets of the particle has to travel long distances back to the indent in order to fill it. It would be much more natural for this additional material to be redistributed in the close vicinity, i.e., at the side facets, and at the top facet close to the particle edge. Indeed, our simulations of the shape evolution of the indented disc‐shaped particle of the dimensions similar to those in Figure 1i demonstrate that classical curvature‐driven surface diffusion alone is incapable to cause the full indent healing, and restoration of the initial particle shape (see Figure S2 of the Supporting Information).

### 2.2 Atomistic Computer Simulations

Although the experimental and the MD simulations timescales differ by orders of magnitude, MD simulations (see details in the Experimental Section) are used to rationalize the essential mechanisms of the observed phenomenon of nanoparticles self‐healing, and to motivate the analytic semiquantitative diffusion model. Or, in other words, the essential structural features of atom movements are extracted from the MD simulations, whereas the kinetics of healing obtained in the MD simulations is not expected to be directly comparable with the experimental data.

**Figure 3:**
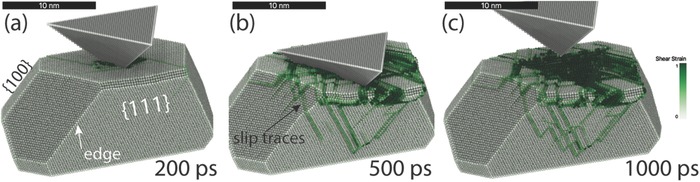
Slip traces in the Au nanoparticle during the nanoindentation in the MD simulation a,b) during the loading and c) upon unloading. The atoms are colored according to the atomic shear strain with the color map from white to green for the shear strain from zero to 1, respectively.

**Figure** **3** visualizes the evolution of the surface during the indentation with an infinite stiff cube‐corner indenter tip. During the indentation, the nucleated dislocations in nanoparticle impinge the free surfaces and Burgers vector is deposited on the free surface by the ledge formation, which eventually results in several surface terraces surrounded by ledges. During deformation, the mass transport from the indented region to the free surfaces is mediated by shear deformation via dislocation loops expanding through the bulk, with the residual signatures on nanoparticle's facets. These slip traces are strictly geometrically connected by Burgers vector conservation to the vicinity of the region of dislocations nucleation (i.e., indent imprint). In other words, the origin of the mechanically induced mass transport is predominately encoded in the network of slip traces on the facets (green traces in Figure 3c), without significant residual dislocation network in the nanoparticle. We also observed that a single nucleation site can be responsible for several dislocation nucleation events, leading to the formation of ledges of multiple heights (in terms of Burgers vector projection on the side facet normal), see Figure 3b,c.

**Figure 4:**
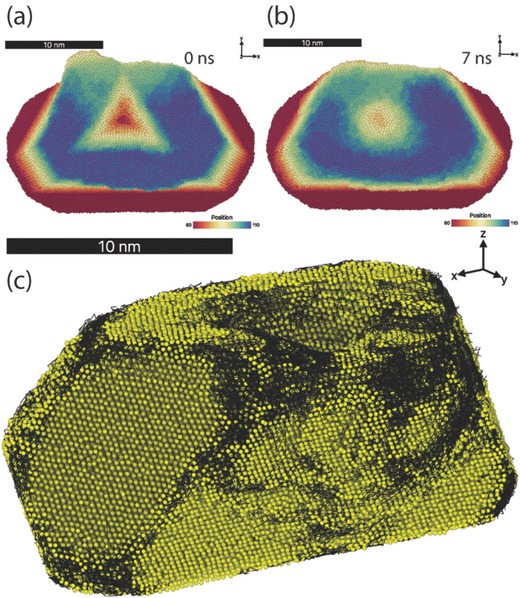
Top view on the indented nanoparticle a) at the beginning and b) during the annealing at 1000 K in the MD simulation. The atoms are colored according the z‐position to reveal the recovery of the initial shape. c) Perspective view on the nanoparticle during annealing with its meandering terrace ledges, which together with the facet edges and the minority {100} facets represent the fast diffusion pathways. The atomic trajectories are indicated by the superimposed black lines and were computed for atoms which moved more than 0.1 nm within 10 ps, to be distinguishable from the thermal fluctuations.

The subsequent annealing at 1000 K shows that the residual indent (**Figure** **4**a,b) recovers toward the initially flat {111} facet, while the overall nonequilibrium shape of nanoparticle remains approximately unaltered. Close inspection of the atomic trajectories (black lines in Figure 4c) reveals atomic diffusion to be predominately along the slip traces, the geometric edges between the facets, and on the minority {100} facets, which appear to be thermodynamically roughened at 1000 K for the interatomic interaction potential employed in the present work. The ledges formed at the slip traces represent the fast diffusion pathway for mass transport (see the Movie S1 of the Supporting Information), which directly guides the Au atoms from the side facets into the residual indent. The latter is bound by the high‐index, high‐energy surfaces which serve as nearly perfect sinks for the atoms diffusing from the adjacent facets.

The fast self‐diffusion along the ledges and edges leads to the shape evolution of the indented nanoparticle toward its global equilibrium shape, as indicated by continuously decreasing potential energy (not shown here). But the diffusion is also kinetically constrained to slip‐traces induced by plastic deformation, and to the geometrical edges of the initially faceted nanoparticle. This network of ledges and edges “guides” the relaxation toward equilibrium through the “bypass road” which returns the particle to its initial shape in the range of times and temperatures considered here. Once the process of self‐healing is completed, further shape changes toward equilibrium become prohibitively difficult.^20^

The evidence of the role played by the ledges during our experiments can be seen in the AFM image of the Au particle close to full recovery in Figure 2f, which remarkably resembles a similar terrace structure obtained in our MD simulations (see the blue atomic layer in Figure 4b). The thermal rounding effect caused by diffusion along the facets' edges can be also observed in the SEM image of recovered particle, see Figure 2d.

### 2.3 Kinetic Model of Diffusion‐Mediated Shape Recovery

To underline the essential features of the atomistic mechanisms uncovered in our MD simulations, we have built a simple model of the diffusion‐mediated indent healing and shape recovery of the particles. We assumed that the dislocations nucleated during indentation of the particle glide sideward and egress at its side facets. The dislocation half‐loop annihilated at the side facet leaves behind a semicircular terrace. Annealing leads to dissipation of the terraces and “filling” of the indent. The dissipation of the terraces is modeled by curvature‐driven diffusion of the atoms along the terrace edges (ledges). This implies that diffusion rate of individual adatoms on the upper surface of the particle is very high, and once the atom diffusing along the ledge emerges on the upper surface, it swiftly finds its way to the indent, which serves as an ideal sink for adatoms. The Ehrich–Schwoebel barrier in Au is assumed to be sufficiently high, such that direct jumps of Au atoms from the ledges to the terraces, and from the side to the upper facet are negligible. The extraordinary stability of nonequilibrium faceted Au particles which were obtained by solid‐state dewetting (and especially of their height) indirectly confirms the large height of Ehrich–Schwoebel barrier.^24^, ^25^ In our MD simulations, we observed very few free adatoms diffusing on the {111} facets, indicating that the direct atomic jumps of Au atoms over the ledge are rare, and confirming the large height of Ehrich–Schwoebel barrier. Also, the direct MD simulations of Na diffusion on terraced surface of Cu performed by Godsi et al. indicate that the height of the Ehrich–Schwoebel barrier is more than twice as high as the energy barrier for atomic jumps along the terraces.^26^ Moreover, we neglected the elastic interactions between the ledges.^27^

We supposed that the equal number of terraces is generated on each side of the particle (*N*_side_), and that all terraces have the same height (*d*_step_). Initially all terraces are semicircular, with the radii *R*_0_ uniformly distributed between 0 and *R*_max_. *R*_max_ is chosen to be ≈1.2*H*_0_, where *H*_0_ is the indentation depth. This implies that initial radius of the *k*‐th terrace (*k* = 0 – *N* – 1) is

(1) $$R_{o}(k)=R_{\max}(1-k/N)$$

The number of the terraces (*N*) on each side of the particle is calculated employing mass conservation

(2) $$N_{\mathrm{side}}d_{\mathrm{step}}\sum_{k=0}^{N-1}0.5\pi R^{2}{}_{\max}{\left(1-k/N\right)}^{2}=V_{0}$$

where *V*_0_ is initial volume of the indent. We assume the cube‐corner geometry of the indent

(3) $$V_{0}=0.5H_{0}^{3}\sqrt{3}$$

**Figure 5:**
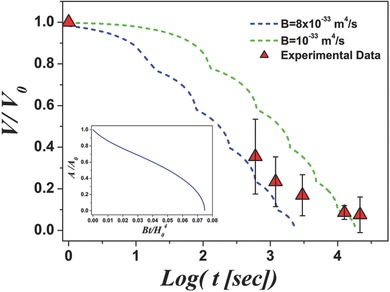
The dependence of the relative area of the single side terrace on dimensionless annealing time (the inset), and the simulated dependencies of the total relative material volume in the side terraces on annealing time (dashed lines), together with the experimental data on the dependence of the relative volume of the indent on annealing time (triangles).

Each terrace evolves with time according to Mullins' equation^10^

(4) $$v_{n}=B\frac{\partial \kappa^{2}}{\partial s^{2}}$$

(5) $${\kappa (O_{1})=\kappa (O_{2})=0,\;\frac{\partial y}{\partial s}|}_{o_{2}}={-\frac{\partial y}{\partial s}|}_{o_{1}}=1,$$

where *v*_n_ is local normal velocity, κ is local curvature, *s* is arc‐length along the ledge, O_1_ and O_2_ are the termini of the ledge on the upper facet, and *B* is the Mullins coefficient describing the self‐diffusion of Au atoms along the ledge

(6) $$B=\frac{D\nu \Omega^{2}\gamma }{kTd_{\mathrm{step}}^{2}}$$

with *D*, ν, Ω, γ being the diffusion coefficient of Au atoms along the ledge, the number of mobile atoms per unit length of the ledge, the atomic volume of Au, and the linear energy of the ledge, respectively. *kT* has its usual thermodynamic meaning. The boundary conditions (5) reflect an assumption that diffusion along upper surface of the particle is so fast that it does not limit mass transfer. The universal temporal evolution of the normalized terrace area *A*/*A*_0_ is shown in the inset of **Figure** **5**. During initial stage the terrace narrows, followed by self‐similar shrinkage with a stabilized shape. The temporal evolution of terrace area *A* can be approximated as

(7) $$A=A_{0}F(Bt/A_{0}^{2}),\;\;F(u)=0.12e^{-100u}+0.88\sqrt{1-13.37u}$$

The total volume of the material in the terraces is calculated based on the Equation (7)

(8) $$V=N_{\mathrm{side}}d_{\mathrm{step}}0.5\pi \sum_{k}R_{0}^{2}(k)F\left(4Bt/\pi^{2}R_{0}^{4}(k)\right)$$

where *R*_0_(*k*) is given by Equation (1). The sum in Equation (8) includes the terraces whose initial radius satisfies the following condition (smaller terraces have annihilated earlier)

(9) $$R_{0}(k)>1.526{\left(Bt\right)}^{1/4}$$

Figure 5 shows the dependence of total volume of the material in the terraces (i.e., material displaced from the indent) on time for the following parameters: *H*_0_ = 100 nm, *N*_side_ = 6, *d*_step_ = 3 nm, *N* = 7, for the *B* values of the 8 × 10^−33^ m^4^ s^−1^ (blue dashed curve) and 1 × 10^−33^ m^4^ s^−1^ (green dashed curve), together with the experimental points (red triangles) for the indents produced with the maximum load of 32 µN. It is instructive to compare the values of *B* for ledge self‐diffusion estimated in the framework of our model with the values of Mullins' coefficient for the surface self‐diffusion in Au, *B*_s_. It can be written in the following form

(10) $$B_{s}=\frac{D_{s}\gamma_{s}\delta \Omega }{kT}$$

where *D*_s_, γ_s_, and δ are the surface self‐diffusion coefficient in Au, the surface energy of Au, and the width of the surface layer with highly mobile surface atoms, respectively. The average surface energy of Au is γ_s_ ≈ 1.4 J m^−2^ and the atomic volume is Ω ≈ 1.69 × 10^−29^ m^3^.^28^ The surface self‐diffusion coefficients in Au reported in the literature are varying by several orders of magnitude. Here we use the reported values of *D*_s_ obtained with the aid of surface scratch method, *D*_s_ = 2.45 × 10^−14^ m^2^ s^−1^,^29^ and combined transmission electron microscopy and scanning tunneling microscopy observations of shape evolution of Au clusters on graphite, *D*_s_ = 4.97 × 10^−15^ m^2^ s^−1^.^30^, ^31^ These works were selected because the respective measurements were performed at relatively low homologous temperatures, and they relied on capillary‐driven shape changes relevant for the present study. The corresponding values of the Mullins's coefficient at 873 K are *B*_s_ ≈ 2.4 × 10^−32^ m^4^ s^−1^ and *B_s_* ≈ 4.9 × 10^−33^ m^4^ s^−1^, respectively. The overlap between the ranges of values for the parameter *B* determined with the aid of our kinetic model, and for the parameter *B*_s_ determined from the independent surface self‐diffusion studies in Au indicates that ledge‐guided surface diffusion represents a viable mechanism of self‐healing of indentation damage in Au particles.

The essence of our analytical model inspired by MD simulations is that the slip lines (ledges) on the side facets of the particle serve as “guide rails” directing the curvature‐driven self‐diffusion of Au to the upper surface. From there, the diffusing atoms arrive at the indented site, which served as a perfect sink for diffusing atoms. This way the material accumulated on the side surfaces can fill the indented site, without concomitant changes of the overall shape of the particle. Both the plastic deformation during the indentation of Au particles and the capillary‐driven diffusion along the ledges during the following healing heat treatment are the classical examples of irreversible processes, and it is fascinating that a combination of two irreversible processes leads to the damage recovery and reversible restoration of the particle shape.

Finally, at the present stage we cannot fully understand why annealing of the deformed Au particles of similar shape and dimensions at higher temperature of 1173 K did result in their equilibration.^15^ One possibility may be related to the residual dislocations left in the deformed particles once the indentation load is relieved. While annealing at the temperature of 873 K employed in the present study leads to a mere recovery of this dislocation structure, annealing at higher temperatures may result in particle recrystallization and formation of one or several grain boundaries. The grain boundaries are the potent sources of steps and are known to accelerate the particle equilibration.^32^ More experiments are needed in order to determine the temperature dependence of the mechanisms of particle shape evolution.

## 3 Conclusions

We demonstrated that the localized plastic deformation of faceted single crystalline Au nano and microparticles produced by sharp diamond tip can be almost fully recovered by annealing the deformed particles at the temperature of ≈0.65*T*_m_, where *T*_m_ is the melting point of Au. The deformed particles restore their initial anisotropic, polyhedral shape which is different from the thermodynamically equilibrium shape of Au crystal of the same volume. Our atomistic MD simulations and kinetic model demonstrated that the mechanism of the observed shape memory and self‐healing effects is related to the accelerated diffusion of Au atoms along the terrace ledges formed by the dislocations egressing at the free surface of the particle during plastic deformation. This allows us to formulate the conditions necessary for observing the shape memory and self‐healing effects in metal nano and microparticles:

1. The plastic deformation of the particles has to be initiated by dislocation nucleation in a defect free crystal without subsequent dislocation storage in the bulk, since all plastic flow of material should be accommodated by the surface slip lines.^22^, ^33^, ^34^ Therefore, the effects observed in the present work are limited to the deformation of faceted metal particles, and annealing below the surface roughening temperature.
2. The full shape equilibration and achieving the thermodynamic equilibrium crystal shape should be suppressed, i.e., by the high energy barrier associated with the island nucleation on the facets.^20^ This indicates a restricted window of parameters (annealing time, temperature, and particle size) for self‐healing.

Prospectively, the demonstrated self‐healing and shape memory effect in metal nano and microparticles could be utilized for the design of mechanically robust and damage‐tolerant components and devices at the submicrometer length scale.

## 4 Experimental Section

*Experimental Details*: The Au particles were obtained employing solid state dewetting of 30 nm thick Au film deposited by the electron‐beam deposition technique on the *c*‐plane oriented polished sapphire substrate of 2 in. in diameter (Gavish Inc.). The substrate was ultrasonically cleaned in acetone, ethanol, isopropanol, and deionized (DI) water and lithographically patterned prior to the deposition. The photolithography procedure included standard process steps: vapor prime with hexamethyldisilazane, spin coating of resist, soft bake, contact printing exposure using mercury lamp source mask aligner (KARL SUSS MA‐6), post bake, development, and hard bake. The liftoff procedure was performed in 70 °C 1‐methyl‐2‐pyrrolidone for 3 min followed by rinsing in acetone, ethanol, isopropanol, and DI water. The samples were annealed in the tube resistance furnace in ambient air for 24 h at 1173 K, resulting in agglomeration of the patterned film and formation of the faceted Au particles.

Some of the obtained particles were indented in the load control mode in AFM (XE‐70, Park systems). The nanoindentation probe employed in the experiments was a pyramidal diamond tip attached to the sapphire cantilever (MicroStar Tech, stiffness constant *k* = 181 N m^−1^). The loading/unloading rate was set at 1.5 µN s^−1^, and several sets of indentations of the particles with different maximum loads were performed.

The characterization of all sites with indented particles was performed by HR‐SEM (Zeiss Ultra Plus) and AFM operated in tapping mode and equipped with sharp Si probes (NSG 30, NT‐MDT, stiffness constants in the range of 22–75 N m^−1^). Full characterization was performed after the indentation, and after each of the following heat treatments. For the HR‐SEM imaging, the samples were mounted onto clamp holders without the use of gluing tapes and without deposition of conducting layers, to avoid contaminations and allow repeated heat treatments. The samples were annealed in ambient air in the same resistance tube furnace that was employed for producing the particles. The annealing temperature was 873 K. The quartz boat with the sample positioned on the clean sapphire wafer was introduced into the hot zone by manipulator, and the heating time to the target temperature was about 4 min.

*Computational Details*: The nonequilibrium initial geometry of the Au nanoparticle was adapted from one of the experimentally observed particles, with (111) surface being parallel to the substrate. The indenter tip was simulated by a rigid cube‐corner shaped diamond lattice, interacting repulsively with the Au nanoparticle. The substrate is modeled by a flat wall with a short‐range Lennard‐Jones interaction with Au atoms to ensure adhesion. The Au–Au interatomic interaction is modeled with the established embedded atom method potential.^35^

Upon relaxation and equilibration at 300 K, the deformation by the indenter tip was induced by displacement controlled loading and unloading with the constant indenter velocity (10 m s^−1^). After mechanical deformation, the nanoparticle on the substrate was annealed at a constant temperature (1000 K), well below the melting temperature of the nanoparticle and the used interatomic potential. All simulations were performed employing the LAMMPS package,^36^ and visualizations and postprocessing was performed with the aid of OVITO package.^37^

## Conflict of Interest

The authors declare no conflict of interest.

## Supporting information

Supplementary
