# Supplementary material for: Self‐Healing and Shape Memory Effects in Gold Microparticles through the Defects‐Mediated Diffusion
Source: Adv Sci (Weinh). 2017 Jul 7;4(8):1700159. doi: 10.1002/advs.201700159 (PMC5566342; doi:10.1002/advs.201700159)
Supplement: Supplementary file 1 — Supplementary [file ADVS-4-na-s001.pdf]

Copyright WILEY-VCH Verlag GmbH & Co. KGaA, 69469 Weinheim, Germany, 2017.

ADVANCED SCIENCE
Open Access

Supporting Information

for *Adv. Sci.,* DOI: 10.1002/advs.201700159

Self-Healing and Shape Memory Effects in Gold Microparticles through the Defects-Mediated Diffusion

*Oleg Kovalenko, Christian Brandl, Leonid Klinger, and Eugen Rabkin**

Copyright WILEY-VCH Verlag GmbH & Co. KGaA, 69469 Weinheim, Germany, 2017.

Supporting Information

Self-healing and shape memory effects in gold microparticles through the defects-mediated diffusion

*Oleg Kovalenko, Christian Brandl, Leonid Klinger, and Eugen Rabkin**

1. The dependence of particles hardness on their aspect ratio

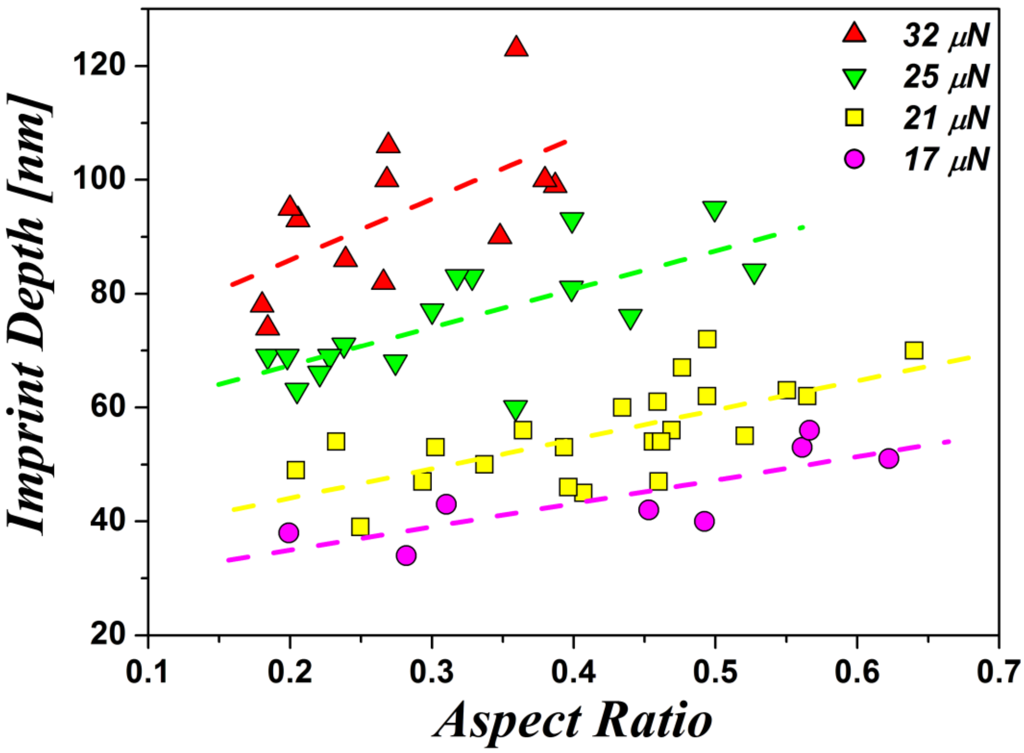

Figure S1. The dependence of indent depths on the aspect ratios for all studies Au particles. The decrease of indentation hardness of the particles with increasing aspect ratio indicates the increasing role of side surfaces as dislocation sinks.[1]

2. Shape evolution of axisymmetric particle due to surface diffusion

We considered a disc-shaped Au particle of initial height of 150 nm, and the radius of 500 nm. We further assumed that the indenter has a conical shape, with the area function of the indenter being identical to that of the cube corner indenter. The result of the indentation is modeled by a conical cavity of 75 nm in depth positioned at the particle axis. All material displaced by the indenter is homogeneously re-deposited at the edge of the upper half of the particle. We assume that the particle shape evolves by isotropic surface diffusion mechanism:

$$v_n = B_s \Delta_s \kappa \qquad \text{(S1)}$$

where κ is surface curvature and Δ_s is the Laplace-Beltrami operator.[2] For calculating the Mullins coefficient B_s we employed the surface self-diffusion data in Au obtained by Göbel, and von Blanckenhagen.[3] The numerical algorithm described earlier was employed for solving Equation (S1).[4] The results are presented in **Figure S2**. One can see that isotropic surface diffusion causes the lateral shrinkage of the particle and significant increase of its height, while the sharp indent evolves into smoothed central depression. These features are very different from our experimental observations indicating full healing of the indent and stability of the particle height. Thus, we can conclude that the isotropic surface diffusion alone cannot cause full indent healing as observed in the present work.

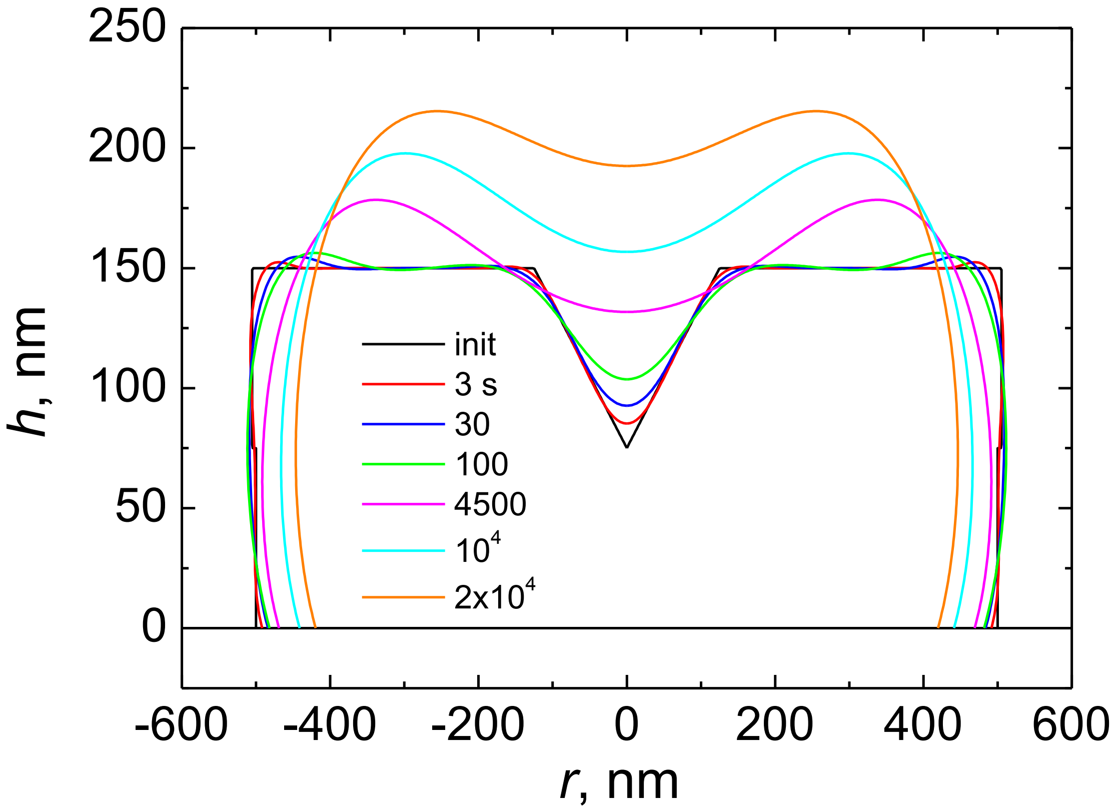

Figure S2. Shape evolution of disc-shaped particle with the central conical cavity mimicking the cube corner indent. The morphology evolution is modeled with the aid of Mullins' equation (S1), with the surface diffusivity determined by the surface scratch method.[3] The annealing times at 873 K are given in the figure.

3. The dynamics of atom movements in MD simulations

Movie M1. The dynamics of atom movements and the particle topography (in the insert) during atomistic MD simulations.

4. References

[1] D. Mordehai, M. Kazakevich, D. J. Srolovitz, E. Rabkin, *Acta Mater.* **2011**, *59*, 2309.
[2] W. W. Mullins, *J. Appl. Phys.* **1959**, *30*, 77.
[3] H. Göbel, R. von Blanckenhagen, *Surf. Sci.* **1995**, *331–333*, 885.
[4] N. Gazit, L. Klinger, E. Rabkin, *Acta Mater.* **2017**, *129*, 300.
